# Tumor Regression Grading of Gastrointestinal Carcinomas after Neoadjuvant Treatment

**DOI:** 10.3389/fonc.2013.00262

**Published:** 2013-10-07

**Authors:** Svenja Thies, Rupert Langer

**Affiliations:** ^1^Institute of Pathology, University Bern, Bern, Switzerland

**Keywords:** tumor regression grade, histopathology, gastrointestinal cancer, neoadjuvant therapy

## Abstract

Multimodal therapy concepts have been successfully implemented in the treatment of locally advanced gastrointestinal malignancies. The effects of neoadjuvant chemo- or radiochemotherapy such as scarry fibrosis or resorptive changes and inflammation can be determined by histopathological investigation of the subsequent resection specimen. Tumor regression grading (TRG) systems which aim to categorize the amount of regressive changes after cytotoxic treatment mostly refer onto the amount of therapy induced fibrosis in relation to residual tumor or the estimated percentage of residual tumor in relation to the previous tumor site. Commonly used TRGs for upper gastrointestinal carcinomas are the Mandard grading and the Becker grading system, e.g., and for rectal cancer the Dworak or the Rödel grading system, or other systems which follow similar definitions. Namely for gastro-esophageal carcinomas these TRGs provide important prognostic information since complete or subtotal tumor regression has shown to be associated with better patient’s outcome. The prognostic value of TRG may even exceed those of currently used staging systems (e.g., TNM staging) for tumors treated by neoadjuvant therapy. There have been some limitations described regarding interobserver variability especially in borderline cases, which may be improved by standardization of work up of resection specimen and better training of histopathologic determination of regressive changes. It is highly recommended that TRG should be implemented in every histopathological report of neoadjuvant treated gastrointestinal carcinomas. The aim of this review is to disclose the relevance of histomorphological TRG to accomplish an optimal therapy for patients with gastrointestinal carcinomas.

## Introduction

Multimodal treatment has been successfully introduced in the therapy of gastrointestinal malignancies. Preoperative chemo- or radiochemotherapy followed by surgery or perioperative treatment currently represents the standard approach for locally advanced esophageal, gastric, and rectal carcinomas, providing survival benefit for the patients compared to surgery alone, and especially patients with complete or subtotal regression of the tumors show significant improved survival rates (1–9).

The effects of neoadjuvant chemo- or radiochemotherapy can be determined by histopathological investigation of the subsequent resection specimens (10–13). Not all tumors show regressive changes in a similar manner. In most cases, complete or subtotal tumor regression after neoadjuvant treatment is associated with better outcome of the patients. There exist many tumor regression grading (TRG) systems which aim to categorize the amount of regressive changes after cytotoxic treatment – mostly they refer to the amount of therapy induced fibrosis in relation to residual tumor (14, 15) or the estimated percentage of residual tumor in relation to the previous tumor site (12, 16–19). Since they have been shown to provide highly valuable prognostic information they may represent a morphological marker for subsequent guiding of patients after neoadjuvant treatment and surgery.

In this review we will present examples of four very commonly used TRG systems for gastrointestinal carcinomas, discuss their clinical and prognostic relevance and also address the limitations and critical issues such as interobserver variability and lack of standardization, finally presenting a personal proposal for an instruction of a standardized macroscopic and histologic work up of resection specimens of neoadjuvant treated gastrointestinal carcinomas.

## Regressive Alterations of the Tumors after Neoadjuvant Therapy

**Figure 1:**
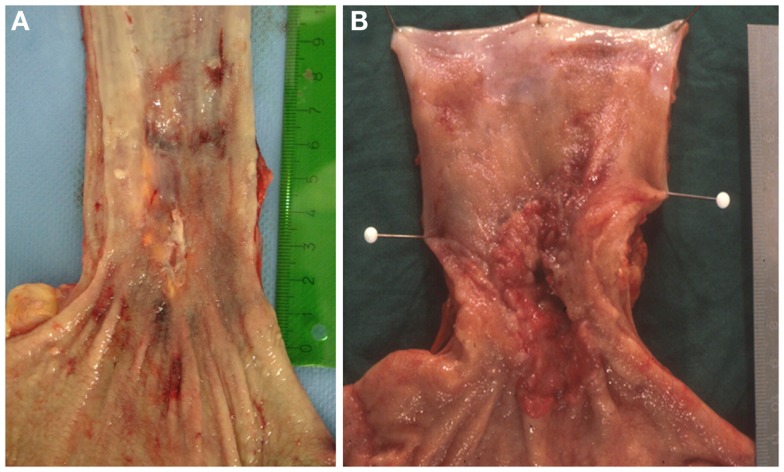
**Gross images of esophageal adenocarcinomas with (A) macroscopic significant regression and (B) no macroscopic significant regression after neoadjuvant chemotherapy**.

**Figure 2:**
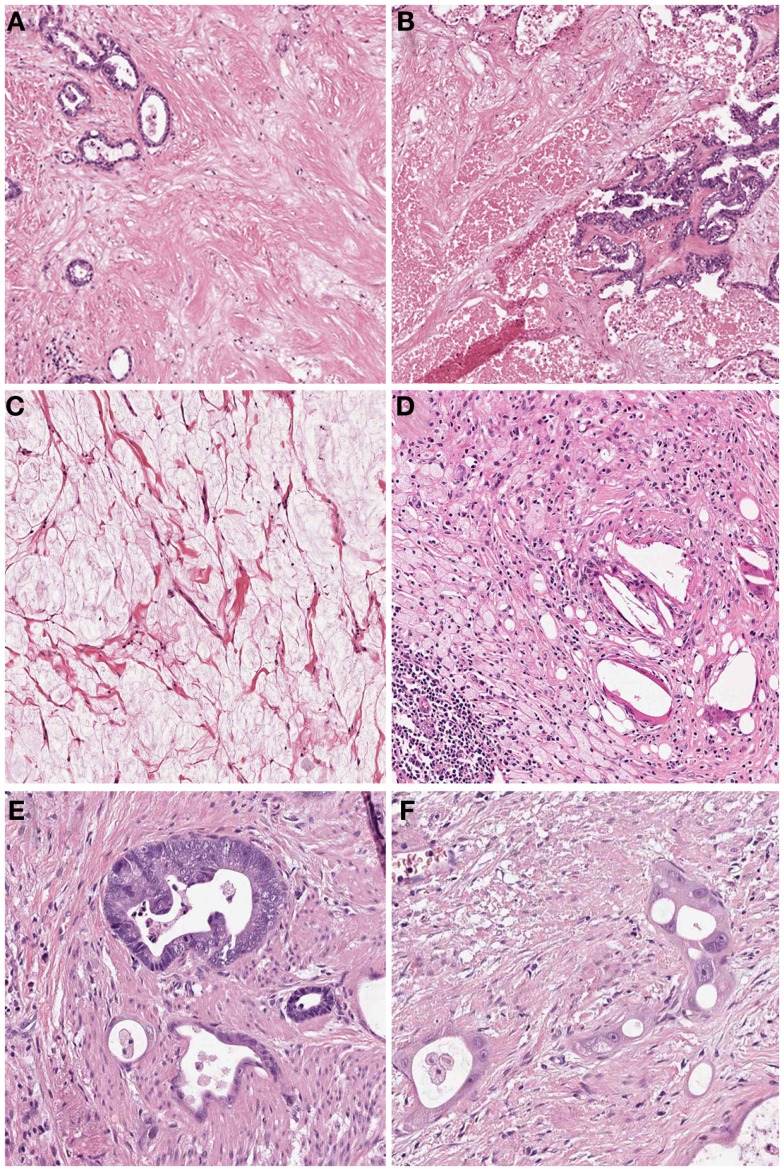
**Histologic findings of tumors treated by neoadjuvant (radio) chemotherapy**. **(A)** Fibrosis replacing previous large parts of the tumor which is evident only by scattered residual tumor glands (Hematoxylin and Eosin (HE) stain, 10×); **(B)** Acute necrosis (HE stain, 10×); **(C)** Acellular mucin lakes (HE, 20×); **(D)** Foamy histiocytes and resorptive changes with cholesterol clefts and chronic inflammation (HE, 20×); **(E)** Regressive tumor gland adjacent to a better preserved viable gland (HE, 25×); **(F)** High grade cellular atypia in regressive tumor glands. Note the intratubular histiocytes as sign of resorption (HE, 40×).

In many cases the pathologist can already roughly estimate the degree of tumor regression by macroscopy (Figure 1). The histologic appearance of regression of these tumors (Figure 2) basically represents a subacute-subchronic inflammation because most tumors are resected after a delay of several weeks after completion of the preoperative treatment.

Significant regressive changes may result in complete disappearance of malignant cells and replacement of the tumor by fibrous or fibro-inflammatory granulation tissue. Signs of resorption, like histiocytic reaction with hemosiderin-laden and foamy macrophages, cholesterol deposits, and foreign body reaction, as well as dystrophic calcifications can be seen (10–12, 20). In this context it should be stressed, that especially the presence of foamy histiocytes has been shown to be most specific for regression due to previous cytotoxic treatment while stromal changes like fibrosis, granulating inflammation following endogenous tumor necrosis can also be observed in untreated carcinomas (12). Another frequent finding of adenocarcinomas treated by neoadjuvant therapy is the presence of acellular mucin lakes (12, 21, 22). They should not be considered as viable residual tumor, however the presence of mucin lakes warrants an intense search for residual vital tumor cells (10). Tumor regression may also follow a centrifugal pattern, and even if the superficial tumor has completely regressed, residual tumor may be found in deeper areas of the tumor bed or at the periphery, respectively (12).

On the cellular level, the residual malignant cells can show eosinophilic cytoplasm, vacuolization of cytoplasm, or undergo oncocytic differentiation. Development of neuroendocrine differentiation may be seen. Marked nuclear atypia with hyperchromasia, karyorrhexis, pyknosis, or enlargement of nuclei with sometimes bizarre formations are frequent findings. Giant cells may also be present. Mitoses are found rarely in contrast to apoptotic figures. These alterations may be quite localized, with histologically typical areas of cancer infiltrates immediately adjacent to marked cytopathic atypical cells (10, 12, 20).

Bizarre stromal fibroblasts can be observed in tumoral and non-tumoral tissue. Vascular alterations like myxohyaline intimal proliferation of vessels, sometimes with highly atypical endothelial cells, telangiectasia, organizing thrombi, and endarteritis obliterans are frequently seen. The non-neoplastic tissue may also undergo treatment associated changes, such as non-tumorous inflammatory ulceration, submucosal edema, and inflammation. Epithelial changes comprise variabilities in nuclear/cytoplasmic ratio, nuclear pleomorphism, condensed chromatin, and the presence of prominent, multiple, and irregular nucleoli. Gland structures of the esophagus and stomach may show atrophy and metaplastic changes (10, 12, 20). All these findings on non-neoplastic tissue may sometimes appear worrisome and can be difficult to distinguish from cancer.

## Classification of Tumor Regression

Tumor regression grading systems aim to categorize the amount of regressive changes after cytotoxic treatment in order to demonstrate potential prognostic information based on objectively determinable histopathologic findings. As stated above, many histopathologically detectable alterations and findings are only infrequently seen and not entirely specific for tumor regression after cytotoxic treatments. Therefore the regression grading systems refer mostly onto single, better reproducible parameters, such as the amount of therapy induced fibrosis in relation to residual tumor or an estimated percentage of residual tumor in relation to the previous tumor site. The TRG systems according to Mandard (15), Becker (12), Dworak (14), or Rödel (17) are examples for commonly used TRGs, which represent different approaches for estimating the degree of tumor regression. An overview about the various TRGs is given in Table 1.

**Table 1:** **Examples for tumor regression grading systems**.

Mandard et al. (15)	Becker et al. (12)	Dworak et al. (14)	Rödel et al. (17)
1. Complete regression (= fibrosis without detectable tissue of tumor)	1a. No residual tumor/tumor bed + chemotherapy effect	0. No regression	0. No regression
2. Fibrosis with scattered tumor cells	1b. <10% Residual tumor/tumor bed + chemotherapy effect	1. Predominantly tumor with significant fibrosis and/or vasculopathy	1. Regression of <25% of tumor mass
3. Fibrosis and tumor cells with preponderance of fibrosis	2. 10–50% Residual tumor/tumor bed + chemotherapy effect	2. Predominantly fibrosis with scattered tumor cells (slightly recognizable histologically)	2. Regression of 25–50% tumor mass
4. Fibrosis and tumor cells with preponderance of tumor cells	3. >50% Residual tumor/tumor bed ± chemotherapy effect	3. Only scattered tumor cells in the space of fibrosis with/without acellular mucin	3. Regression of >50% tumor mass
5. Tissue of tumor without changes of regression		4. No vital tumor cells detectable	4. Complete regression

### TRG according to Mandard

The Mandard classification system was published in 1994 and first applied for estimation of tumor regression in squamous cell carcinomas of the esophagus after neoadjuvant treatment with cisplatin and radiotherapy (15). In this study 93 resected specimens were examined. First, the macroscopic impression was divided into three macroscopic groups: obvious residual tumor with ulceration/fungating/infiltrative feature, apparent tumor regression and scarring, and the last group included doubtful cases. On histology the specimens were separated into two groups with or without regressive changes, while the regressive changes included the stromal changes and cytological alterations. Basing on these changes the tumor regression was classified into five histological TRGs (Table 1), based on vital tumor tissue at the ratio of fibrosis: TRG 1 was defined as complete regression (=fibrosis without detectable tissue of tumor); TRG 2 was defined as fibrosis with scattered tumor cells; TRG 3 was fibrosis and tumor cells with preponderance of fibrosis; TRG 4 was fibrosis and tumor cells with preponderance of tumor cells; TRG 5 was tissue of tumor without changes of regression. Forty-two percent of the tumors were TRG 1–2, 20% were TRG 3, and 33% were TRG 4–5. A high correlation of the disease-free survival (DFS) with the TRG, tumor size, lymph node status, and esophageal wall involvement was seen in univariate analysis. In multivariate analysis, only tumor regression (TRG 1–3 vs. TRG 4–5) remained significant predicator of DFS. In the following years this TRG system was widely applied on each kind of gastrointestinal cancers after neoadjuvant treatment rendering it one of the most widely used TRGs (10).

### TRG according to Becker

**Figure 3:**
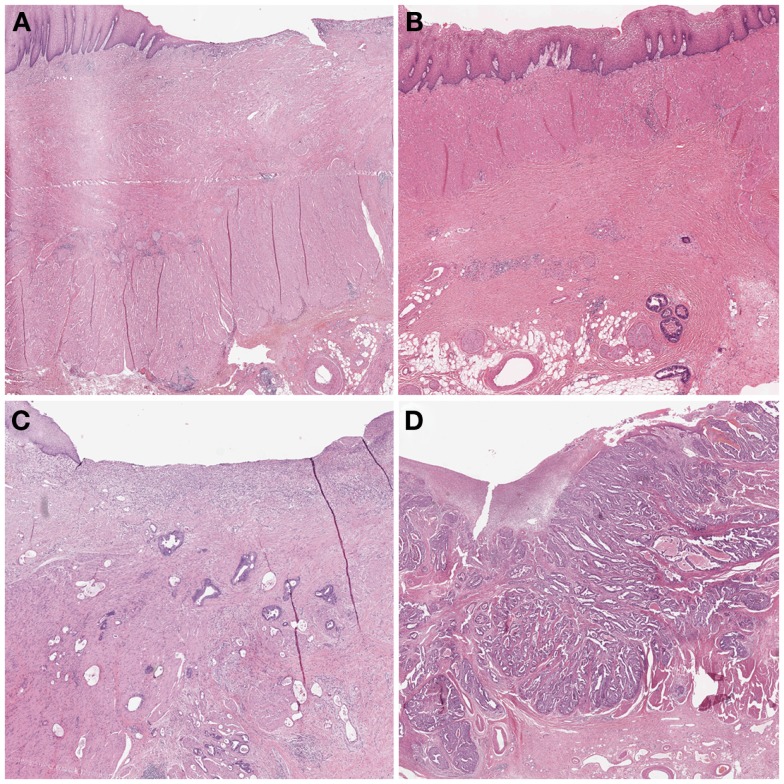
**Examples of tumor regression grades according to Becker**. **(A)** TRG 1a complete regression. This image would be classified as TRG 1 according to Mandard and TRG 4 according to Dworak. **(B)** TRG 1b <10% residual tumor. Mandard TRG would be 2, Dworak TRG 3. **(C)** TRG 2 10–50% residual tumor. Mandard TRG would be 3, but 2 could also be possible since there is no strict definition of “scattered tumor cells” and “preponderance of fibrosis”; Dworak TRG would be 2: the term “scattered tumor cells” is by complemented by “histologically slightly recognizable.” **(D)** TRG 3 >50% residual tumor. Mandard TRG would be 4 or 5 and Dworak 0 or 1 since one can appreciate fibrotic stands which could be preexisting desmoplasia or regression.

In 2003, Becker et al. (12) proposed a different grading system for advanced gastric carcinomas treated by cisplatin based neoadjuvant chemotherapy. In this study, 36 patients with gastric carcinoma were treated neoadjuvant with combined chemotherapy (etoposide, doxorubicin, and cisplatin). The entire macroscopically identifiable residual tumor areas of the specimens and the scarring area were investigated histologically and compared with specimens treated only with surgery alone. The grading of the tumor regression was based on the estimation of the percentage of vital tumor tissue in relationship to the macroscopically identifiable tumor bed (previous site of the tumor) and divided into three grades (Table 1). TRG 1a was defined as complete tumor regression without residual tumor; TRG 1b was defined as <10% residual tumor per tumor bed, like a subtotal tumor regression. TGR 2 illustrated a partial tumor regression with 10–50% residual tumor and at the findings of >50% residual tumor cells with or without signs of treatment effect the tumor regression was classified as TRG 3 (Figure 3). In this study none of the 36 patients had complete tumor regression, 4 patients had TRG 1b, 9 patients had TRG 2 and 23 patients had TRG 3 with more than 50% vital tumor cells. Similar to Mandard, the TRG correlated significantly with survival, besides tumor size and lymphatic vessel invasion. After its description the Becker system was successfully applied in other gastrointestinal malignancies (23, 24). In 2011 the same group confirmed the findings of the initial study by analyzing 480 gastric carcinomas rendering TRG as independent prognostic factor for gastric cancer besides postoperative lymph node status (25).

A large number of other regression grading systems use the principle of the estimation of residual tumor for classification of TRGs: the Japanese Society for Esophageal Disease designate tumor regression as a continuous variable and categorized into four groups as a measure of the extent of chemoradiation response: ypV_0_, no viable cell; ypV_1_, 1–33% viable tumor; ypV_2_, 34–66% viable tumor; and ypV_3_, 67–100% viable tumor (26). The regression grading to Schneider for esophageal cancer is a four step-grading system basing on the percentage of vital tumor cells using the same cut-offs like the Becker system (16). The British Royal College of Pathologists describes a modified grading system with the following cut-offs: 0–5, 5–50 and >50% residual tumor (27).

### TRG according to Dworak

For rectal cancer, in 1997 the group of Dworak et al. (14) described their TRG system basing on the findings on 17 patients who had received preoperative radiochemotherapy with 5-FU/50 Gy. According to the macroscopic features different techniques of sampling of the tumor tissue were applied. If no visible tumor tissue was present the whole suspect area of fibrosis was sliced and embedded. For macroscopically obvious tumor, a minimum of four paraffin blocks was processed and an additional large area block was embedded. The grading of regression was established as follows (Table 1): TRG 0 tumor without regression, TRG 1 with dominant tumor mass with obvious fibrosis and/or vasculopathy. TRG 2 shows dominantly fibrotic changes with few tumor cells or groups, easy to find, while TRG 3 describes only very few tumor cells, difficult to find, in the fibrotic tissue with/without mucous substance. For TRG 4 only fibrotic mass without tumor cells exist, i.e., total regression or response. Although based on a very small sample size, the Dworak system is now widely applied on rectal cancer and also recommended by several national guidelines (28).

### TRG according to Rödel

In 2005, Rödel et al. (17) have used a five tiered score for the estimation of tumor regression in rectal cancer ranging from grade 0 (no regression) to grade 4 (complete regression) in <25, 25–50, and >50% intervals which do not describe the amount of residual tumor but the degree of tumor regression (Table 1). In their study, complete regression was observed in 10% of the cases and an intermediate pathologic response (TRG 2 and 3) could be detected in 65%. Complete and intermediate pathologic response suggested improved DFS after preoperative radiochemotherapy and surgery. For esophageal cancer, Rizk et al. have been using a similar approach by estimation the degree of tumor regression instead of residual tumor. They defined six grades (0–20% regression; 20–50% regression; 50–80% regression; 80–90% regression; 90–99% regression; 100% regression) (19).

## Prognostic Significance of TRG

There are numerous studies which show the prognostic relevance of TRGs. For upper gastrointestinal cancers there is the strongest evidence for the association between TRG and patient outcome. There is the general observation that patients with complete tumor regression do best (10, 25). For esophageal squamous cell and adenocarcinomas some studies could also demonstrate a benefit for patients with subtotal and partial tumor regression (i.e., Becker 1b and 2; Mandard 2 and 3), supporting a distinct biology of esophageal carcinomas (6, 11). For gastric cancer, in contrast, there seems to be a clear discrimination into responding patients with complete and subtotal regression (i.e., Becker 1a, b; Mandard 1 and 2) and non-responders with partial and no regression (i.e., Becker 2 and 3; Mandard 3–5) (25). Interestingly an association between tumor localization and tumor type and TRG could be shown, more distal cancers of Laurens diffuse type having the lowest probability for showing significant tumor regression after neoadjuvant chemotherapy (29).

For rectal cancer, two works have also provided convincing evidence to support TRG as an independent predictor of survival (7–9). Patients with pathological complete regression showed improved disease-free survival, lower risk of local recurrence, better chance of being free from distant metastasis and increased overall survival. TRG, especially in terms of complete regression, therefore is considered to representing a potential tool to guide therapy in patients with rectal cancer as well (30). Additionally, in a very recent meta-analysis, partial tumor regression was described as being associated with improvement in DFS and was therefore also considered as favorable prognostic factor (31).

The prognostic value of TRG may even exceed those of currently used staging systems (e.g., TNM staging) which are originated from data from untreated tumors. The AJCC (32) but not the UICC (33) considers TRG as additional prognostic factor for rectal carcinomas after preoperative treatment, but do not integrate this into a defined staging system. Therefore, several authors have proposed alternative staging systems incorporating tumor regression grades for carcinomas treated by neoadjuvant therapy in order to provide better prognostication for the patients who had undergone preoperative treatment before surgery (16, 18, 34). However, although promising, these proposed prognostic classifications have not entered clinical practice yet.

## Critical Issues of TRG

The most critical issues regarding the histopathologic estimation of a tumor regression grade are inter- and intra-observer variability and the lack of standardization. These two factors may be the reason for sometimes marked differences in reporting the prognostic value of TRGs which is evident especially for rectal cancers.

Recently, Chetty et al. (35) investigated the level of concordance among expert gastrointestinal pathologists for regression grading in rectal cancer treated with neoadjuvant chemoradiation. In this study seventeen pathologists applied the Mandard, the Dworak, and the modified Royal College of Pathologists (mRCP) regression grading systems on selected slides of 10 tumors. Only in one of ten cases did all 17 participants agree concordantly. The Mandard and Dworak grading systems had unsatisfactory interobserver-agreement with kappa values of 0.28 and 0.35, respectively. The mRCP system which bases on the estimation of residual tumor in percentage showed slightly better kappa values with overall kappa-score of 0.38. The study also contained a questionnaire for the contributing pathologists covering several aspects of TRG. The paper concluded that there was a need for a simple, reproducible regression grading system with clear criteria. Moreover, the authors recommended a cumulative or composite score taking into account all sections of the tumor bed that is sampled rather than the worst section and finally a uniform method of sampling of these specimens. Other studies, in contrast, demonstrate a good reproducibility of TRGs; however, they show that the most frequent source of disagreement was assessment of the relative amount of fibrosis, while displacement of epithelium or the misinterpretation of acellular mucin was a minor source of disagreement (36–38).

A very recent study by Mirza et al. (39) compared three TRG systems (Mandard, Japanese Society for Esophageal Disease and Becker) based on their reproducibility and ability to predict survival for gastric carcinomas and adenocarcinomas of the gastro-esophageal junction after neoadjuvant chemotherapy. Sixty-six cases were reviewed by two histopathologists. The highest kappa-score was achieved for the grading system described by Becker (kappa-score = 0.52). The Mandard grading system achieved a kappa-score of 0.44, while the Japanese Grading system had the lowest score with 0.28. While both Mandard and Becker TRGs were associated with patient prognosis, the Becker system was the most reproducible system, and the usage of the percentage of viable tumor cells as criterion of response appeared to be more easily and reproducibly identifiable than the use of the degree of fibrosis. Similar results with respect to the reproducibility of TRG assessment using percentage of residual tumor have also been presented in a study of Wu et al (36).

## Standardization of Work Up and Reporting TRG

Currently there are only few published recommendations for handling of surgical resection specimens although recent papers have demonstrated the strong demand for the implementation of standardization in this field (30, 40). Some authors consider standard processing protocols used for routine cases as appropriate if tumor is grossly visible (10). However, there is general agreement if viable tumor is not grossly evident, embedding the whole suspicious area with the application of step sectioning should be performed (10). For the reliable assessment of complete tumor regression in rectal cancer for example, the second European Rectal Cancer Consensus Conference recommended to take initially at least five tissue blocks from the tumor site, and the whole tumor area should be blocked only if there is no viable tumor. They consider the diagnosis of complete pathological response as appropriate if after additional three levels step sections still no tumor is present (41).

The authors of the present paper would recommend the embedding of the entire tumor bed from the beginning. Small residual tumor infiltrates in the periphery, which is a frequent finding, cannot be detected when the tumor bed is not entirely blocked for histopathological analysis. In cases where the tumor bed measures more than 8 cm in largest dimension, significant regression is unlikely and taking blocks following the longitudinal and vertical largest dimension can be considered as sufficient. If then no or less residual tumor is detected, complete embedding of the remaining tumor bed can be performed in a second step. Moreover, following own experience and supported by the findings of others (36, 39) the authors favor the four tiered Becker system basing on percentage of residual tumor over TRGs which base on the estimation of fibrosis in relation to residual tumor. A personal proposal for a standardized grossing and histopathological reporting is provided in Table 2.

**Table 2:** **Personal proposal for standardized work up and reporting of TRG [modified from Ref. (11)]**.

**PHOTOGRAPHIC DOCUMENTATION**
Photocopy or photograph of resection specimen (orientation and documentation of blocks and of histologically proven residual tumor)
Macroscopic description; tumor size (three-dimensional), distance to resection margins
**WORK UP**
Inking of the deep (circumferential) resection margin
Complete embedding of the macroscopically identifiable tumor bed, orientated from proximal to distal in 0.5 cm levels. If tumor bed >8 cm, significant regression in unlikely: first take blocks following the longitudinal and vertical largest dimension. If no or less residual tumor embed remaining tumor bed in second step. CRM is included in these blocks
All slides stained by Hematoxylin/eosin, selected blocks by periodic acid-schiff, Elastica van Gieson staining; immunohistochemistry may be helpful for discrimination of histiocytes and alterated tumor cells
If no residual tumor: another three step sections to confirm complete response
Resection margins oral, aboral
Additional macroscopic findings
Lymph node stations. Immunohistochemistry (pan-cytokeratin) if ypN_0_
**PATHOLOGICAL REPORT SHOULD INCLUDE**
UICC ypTNM status (including L, V, Pn)
UICC R-status
Distance to circumferential resection margin (esophagus; rectum)
Grading, typing (according to WHO; additionally Lauren’s type for upper GI adenocarinomas)
Histopathological tumor regression grade (e.g., Becker TRG 1a, 1b, 2, 3)

## Conclusion

In summary, assessing tumor response to neoadjuvant treatment has been shown to be feasible by histopathological examination of the resected specimens in gastrointestinal carcinomas. Therefore, it is highly recommended that TRG should be implemented in every histopathological report of neoadjuvant treated gastrointestinal carcinomas. As mentioned above, e.g., the Mandard or the Becker systems have been successfully applied on tumors different from the site of original description. However, an internationally accepted robust system for the grading of tumor regression in gastrointestinal malignancies following neoadjuvant chemoradiotherapy is still required. Currently existing weaknesses such as interobserver variability may be reduced by individual and institutional training (e.g., participation on ring trials). Both the pathologists’ and clinicians’ community have to work on standardization of specimen processing and reporting of TRGs. With regard to the various TRG systems, it may be a major challenge for an international and interdisciplinary commission to find a consensus on TRG reporting. For the future, however, this may be the prerequisite for generating reliable, evidence based data regarding the prognostic impact of TRG finally rendering it as powerful prognostic morphologic biomarker which then also should be integrated part of clinical staging systems. This may help with clinical decision-making, influence surgical strategies, postoperative adjuvant therapy, and surveillance intensity.

## Conflict of Interest Statement

The authors declare that the paper was conducted in the absence of any commercial or financial relationships that could be construed as a potential conflict of interest.
